# The Size–Growth Rate Relationship in Hepatocellular Carcinoma

**DOI:** 10.1002/jgh3.70224

**Published:** 2025-07-14

**Authors:** Dhanushan Gnanendran, Adina Olaru, Meetal Shah, Jonathan Jackson, Suresh V. Venkatachalapathy, Aloysious D. Aravinthan

**Affiliations:** ^1^ Nottingham Digestive Diseases Centre, Translational Medical Sciences, School of Medicine, University of Nottingham Nottingham UK; ^2^ NIHR Nottingham Biomedical Research Centre, Nottingham University Hospitals NHS Trust and University of Nottingham Nottingham UK; ^3^ Department of Radiology Nottingham University Hospitals NHS Trust Nottingham UK

**Keywords:** liver imaging, liver tumors, malignant

Hepatocellular carcinoma (HCC), a leading cause of cancer‐related deaths worldwide, remains a significant clinical challenge, contributing to ~800 000 deaths each year [1]. Surveillance is an effective strategy in managing HCC, with studies showing improved early‐stage detection, curative treatment, and better survival rates with surveillance [2].

The HCC surveillance strategy includes an ultrasound scan (US) every 6 months, based on the shortest mean doubling time observed in growth kinetics studies conducted in the 1980s [3, 4], a finding that has been corroborated by more recent research [5, 6]. Based on this evidence, international organizations such as the European Association for the Study of the Liver (EASL), along with national bodies, recommend a six‐monthly US for HCC surveillance [2, 3, 4, 5, 6, 7]. However, one issue with this recommendation is the assumption that HCC grows at a relatively predictable and constant rate. Studies show a significant variation in tumor volume doubling time, ranging from 2.2 to 11.3 months [8].

One factor likely to influence HCC growth rate is tumor size. Understanding the relationship between tumor size and growth rate can aid in treatment planning and prioritizing patients at risk of faster tumor progression. To explore this potential association, a prospective study was designed at a single tertiary‐care centre, with approval from the Nottingham University Hospital Clinical Effectiveness Board (19‐223C). All newly diagnosed HCC patients were eligible for inclusion if they had undergone at least two cross‐sectional imaging studies of the same modality (CT or MRI) at different time points prior to any treatment. Patients with a prior history of HCC presenting with recurrent disease were excluded. HCC was defined as any lesion classified as Liver Imaging Reporting and Data System (LI‐RADS) LR‐5 on initial or subsequent cross‐sectional imaging, or confirmed histologically. All cross‐sectional images were independently reviewed by two hepatobiliary radiologists (M.S. and J.J.), showing excellent interobserver agreement (*R* ^2^ = 0.8339). The HCC growth rate was calculated by measuring the maximum diameter on initial and follow‐up cross‐sectional imaging, expressing the change in size over the time interval between the scans.

A total of 144 HCC lesions from 65 patients were included in the analysis. Analysis revealed a significant correlation between initial tumor size and monthly growth rate (Spearman's rank correlation coefficient (ρ) = 0.526, *p* < 0.001). When stratified by initial size, tumors with a diameter ≥ 110 mm exhibited a significantly lower median growth rate compared to tumors with a diameter < 110 mm (median 1.4 [IQR 1.1–2.3] vs. 1.6 [IQR 0.8–3.0] mm/month; *p* = 0.03).

Tumors measuring 10–20 mm exhibited a median growth rate of 0.24 mm per month (IQR 0.12–0.58). At this rate, a 10 mm tumor is expected to double in size in ~2.89 months (IQR 1.20–5.78). Similarly, for tumors in the 20–40 mm range, the median growth rate was higher at 0.43 mm per month (IQR 0.18–1.02), suggesting a more rapid proliferative phase. Accordingly, a 20 mm tumor is expected to double in size within 1.61 months (IQR 0.68–3.85).

A scatter plot of initial lesion size against growth rate revealed a steady increase in growth rate with increasing initial size up to 100 mm. Beyond this, the growth rate plateaued and subsequently declined after exceeding 120 mm (Figure 1), potentially reflecting changes in tumor biology and/or constraints in vascular supply.

**FIGURE 1:**
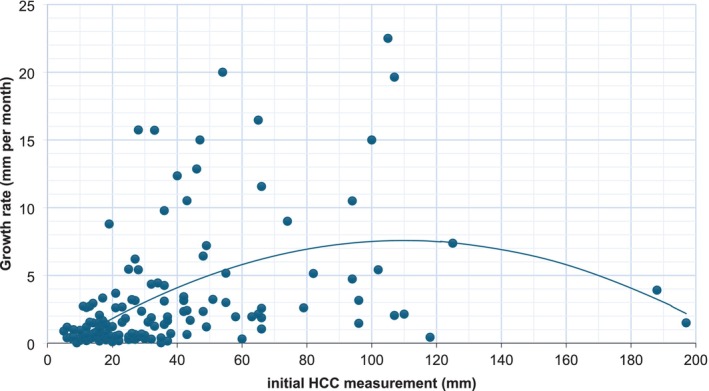
Scatter plot illustrating the relationship between initial HCC size and growth rate.

The findings suggest a logarithmic growth pattern, with rapid early expansion transitioning to slower growth as tumor size increases. This growth trajectory may amplify lead‐time and length‐time biases, potentially diminishing the perceived benefits of HCC surveillance. It remains uncertain whether this pattern is influenced by alterations in mutational burden and tumor biology, changes in blood supply due to increased tumor burden (e.g., the tumor outgrowing its vascular supply), or an artifact of measurement bias, where small differences in tumor diameter have a proportionally greater impact when tumors are smaller.

As this study was based on real‐world data, a proportion of patients did not undergo tumor biopsy, with diagnoses established radiologically in accordance with LI‐RADS criteria. Consequently, it was not possible to assess the impact of other relevant factors known to influence HCC growth, such as histological grade and genetic mutations. In addition, the sample size limited the inclusion of multiple variables in the analysis. Given these constraints, tumor size was selected as the primary variable, as it remains a key determinant in size‐based treatment decisions. Furthermore, given the relatively small number of lesions exceeding 120 mm in the cohort, conclusions regarding growth patterns at advanced stages should be interpreted with caution.

Understanding of tumor growth patterns, particularly in relation to initial tumor size, is critical for informing several treatment decisions. These include the necessity of bridging therapy while awaiting liver transplantation, prioritizing size‐dependent interventions such as ablation or liver transplantation, and determining the optimal surveillance interval for suspicious but non‐definitive lesions or very‐early stage HCCs [9]. This understanding may also aid in predicting the response to HCC treatments, such as trans arterial chemoembolization [10].

Variations in HCC progression may stem from genetic factors, the underlying etiology of liver disease, and associated comorbidities, potentially limiting the universal applicability of these findings. Furthermore, this study does not account for differing growth patterns in patients with recurrent HCC, which may differ from those observed in primary tumors. Future research involving a larger cohort, particularly including patients with initial HCC measurements exceeding 120 mm, could provide further insights into this relationship.

## Conflicts of Interest

The authors declare no conflicts of interest.

## References

[1] M. Arnold , C. C. Abnet , R. E. Neale , et al., “Global Burden of 5 Major Types of Gastrointestinal Cancer,” Gastroenterology 159, no. 1 (2020): 335–349. 32247694 10.1053/j.gastro.2020.02.068 PMC8630546

[2] A. G. Singal , E. Zhang , M. Narasimman , et al., “HCC Surveillance Improves Early Detection, Curative Treatment Receipt, and Survival in Patients With Cirrhosis: A Meta‐Analysis,” Journal of Hepatology 77, no. 1 (2022): 128–139. 35139400 10.1016/j.jhep.2022.01.023 PMC9232881

[3] M. Yoshino , “Growth Kinetics of Hepatocellular Carcinoma,” Japanese Journal of Clinical Oncology 13, no. 1 (1983): 45–52. 6187947

[4] J. C. Sheu , J. L. Sung , D. S. Chen , et al., “Growth Rate of Asymptomatic Hepatocellular Carcinoma and Its Clinical Implications,” Gastroenterology 89, no. 2 (1985): 259–266. 2408960 10.1016/0016-5085(85)90324-5

[5] V. Santi , F. Trevisani , A. Gramenzi , et al., “Semiannual Surveillance Is Superior to Annual Surveillance for the Detection of Early Hepatocellular Carcinoma and Patient Survival,” Journal of Hepatology 53, no. 2 (2010): 291–297. 20483497 10.1016/j.jhep.2010.03.010

[6] J. C. Trinchet , C. Chaffaut , V. Bourcier , et al., “Ultrasonographic Surveillance of Hepatocellular Carcinoma in Cirrhosis: A Randomized Trial Comparing 3‐ and 6‐Month Periodicities,” Hepatology 54, no. 6 (2011): 1987–1997. 22144108 10.1002/hep.24545

[7] European Association for the Study of the Liver , “EASL Clinical Practice Guidelines: Management of Hepatocellular Carcinoma,” Journal of Hepatology 69, no. 1 (2018): 182–236. 29628281 10.1016/j.jhep.2018.03.019

[8] P. Nathani , P. Gopal , N. Rich , et al., “Hepatocellular Carcinoma Tumour Volume Doubling Time: A Systematic Review and Meta‐Analysis,” Gut 70, no. 2 (2021): 401–407. 32398224 10.1136/gutjnl-2020-321040 PMC7657990

[9] N. Mehta , M. Sarkar , J. L. Dodge , N. Fidelman , J. P. Roberts , and F. Y. Yao , “Intention to Treat Outcome of T1 Hepatocellular Carcinoma With the “Wait and Not Ablate” Approach Until Meeting T2 Criteria for Liver Transplant Listing,” Liver Transplantation 22, no. 2 (2016): 178–187. 26479422 10.1002/lt.24360 PMC4803445

[10] Y. Purcell , R. Sartoris , V. Paradis , et al., “Influence of Pretreatment Tumor Growth Rate on Objective Response of Hepatocellular Carcinoma Treated With Transarterial Chemoembolization,” Journal of Gastroenterology and Hepatology 35, no. 2 (2020): 305–313. 31369166 10.1111/jgh.14816

